# Flavonoids: From Structure to Health Issues

**DOI:** 10.3390/molecules22030477

**Published:** 2017-03-17

**Authors:** Celestino Santos-Buelga, Arturo San Feliciano

**Affiliations:** 1 Grupo de Investigación en Polifenoles (GIP-USAL), Universidad de Salamanca, Facultad de Farmacia, Campus Miguel de Unamuno, E-37007 Salamanca, Spain; 2 Grupo de Diseño y Obtención de Moléculas Bioactivas (DOMOBIO-USAL), Universidad de Salamanca, Facultad de Farmacia, CIETUS, IBSAL, AFARCyL, Campus Miguel de Unamuno, E-37007 Salamanca, Spain; asf@usal.es

Flavonoids are one of the largest groups of plant secondary metabolites. They are synthesized through the shikimate/phenylpropanoid and acetate/malonate pathways and comprise compounds that possess a common C6-C3-C6 skeleton, where two aromatic rings are linked through a three-carbon bridge, which usually configures a phenylchromane arrangement. Based on the degree of unsaturation and the substitution pattern, different flavonoid classes are distinguished: flavones, flavonols, flavanones, flavan-3-ols, anthocyanins, dihydroflavonols, and isoflavones, as well as the biogenetic intermediate chalconoid forms. In natural sources, they may occur in free forms (aglycones), as glycosylated or acylated derivatives, and as oligomeric and polymeric structures such as the flavan-3-ol-derived condensed tannins (or proanthocyanidins). Several thousand flavonoids have been identified in plant sources, and the number of compounds increases when considering the flavonoid-derived products that can be formed during the processing and storage of foodstuffs, and the metabolites and conjugates produced in the human organism after their intake. Thus, the chemical nature, structural complexity, physicochemical behaviour, and concentrations of flavonoids notably vary depending on the matrix, making their analysis challenging.

There is no extraction protocol that suits all types of flavonoids and matrices, so sample preparation and extraction conditions usually need to be optimised depending on the nature of the sample, target analytes, and aim of the study. In the present special issue, Zhang et al. [1] established the optimum conditions for maximum recovery of total flavonoids from *Ampelopsis grossedentata* leaves using ultrasonic-assisted solvent extraction and response surface methodology. Further, the main flavonoids of the sample were separated and purified on a large-scale by High Speed counter current Chromatography (HSCCC) for use as analytical standards for quality control of the herb. A sample preparation procedure based on the formation of flavone–metal complexes was employed by Sun et al. [2] for the selective extraction of flavones with 5-hydroxyl or *ortho*-hydroxyl groups from plant and dried blood spot samples, thus avoiding interferences from other background substances in further HPLC analysis.

High purity compounds are usually required for identification by spectroscopic techniques, as well as for study of their physicochemical and bioactive properties. Solvent extraction with further fractionation by column chromatography on different resins was used by Jung et al. [3] for the isolation of isoprenylated flavonoids from the root bark of *Morus alba* (some of them not previously reported) that were identified based on their spectroscopic data, using nuclear magnetic resonance, mass spectrometry, infrared, and circular dichroism techniques. The same strategy was employed by Li et al. [4] to isolate up to sixteen flavonoids from the aerial parts of *Rosmarinus officinalis*, including some new ones, which were further checked for their inhibitory effects on fat accumulation in HepG2 cells. Similarly, five flavonoids were isolated and fully identified from *Paeonia ostii* flowers, used in Chinese folk medicine, and assayed for their in vitro antioxidant activity by Zhang et al. [5].

The preparation of pure compounds by chemical synthesis is an approach especially suitable when relevant amounts are required and/or their efficient isolation from natural sources is difficult. A procedure for the convenient synthesis of naturally occurring minor homoisoflavonoids was described by Lee et al. [6], allowing their preparation for structure–activity relationship studies regarding their antiangiogenic properties. Synthetic flavylium derivatives were obtained by Basilio and Pina [7] to study the chemical and photochemical properties of anthocyanin pigments. Equilibria among structural forms as a function of pH were comprehensively explored using thermodynamic and kinetic approaches. Rates of inter-conversion among forms were found to be influenced by the existence of low or high *cis–trans* isomerization barrier of the chalcone forms, and its level was closely related with the substitution pattern of the flavylium cation.

Mass spectrometry (MS) is commonly employed for the structural characterization of flavonoids. However, it does not provide unequivocal identification of compounds—especially when considering regio- and stereoisomers regarding location and configuration of substituents on the flavonoid molecule, sugars identity, or stereochemistry of glycosidic linkages in oligosaccharides. Some of these gaps can be overcome with high-resolution mass spectra (HRMS), which offer greater mass accuracies and allow obtaining information about elemental composition of the target compounds, as well as by combining several MS techniques (e.g., different systems of ionization, analysis of both positive and negative ions, molecules fragmentation, or addition of metal ions before ionization and fragmentation). The application of these approaches to the identification of flavonoids has been reviewed by Kachlicki et al. [8].

High-performance liquid chromatography (HPLC) combined with different detection systems—especially diode array spectrophotometry (DAS) and MS—is the preferred technique for flavonoid analysis. In recent times, the introduction of technical developments such as novel packing materials for columns, ultra-HPLC (UHPLC) equipment, or coupling to HRMS analysers has provided further improvements in resolution, efficiency, or speed of separation, increasing the chances for compound identification. In this special issue, several works using HPLC-MS approaches for flavonoid profiling in different plant sources are included. Marczak et al. [9] employed UHPLC combined with a Quadrupole Time-of-Flight mass spectrometer (Q-ToF) for the identification of a range of flavonoid conjugates in the medicinal herb *Axyris amaranthoides*. UHPLC coupled to electrospray ionization-mass spectrometry (ESI-MS^n^) was used by Mena et al. [10] to analyse the phytochemical profile of a rosemary (*Rosmarinus officinalis* L.) extract, allowing them to identify and quantify up to 57 compounds, of which 24 were flavonoids. Cuadrado-Silva et al. [11] performed a targeted study of the phenolic constituents of sour guava fruits (*Psidium friedrichsthalianum* Nied.) by UPLC with triple quadrupole mass spectrometry detection (UPLC-ESI/QqQ) in multiple-reaction monitoring (MRM) mode and related them with the antioxidant properties of the fruit. Similarly, Garcia-Ruiz et al. [12] explored the phytochemical composition (flavonoids and carotenoids) of dried and microencapsulated preparations from banana passion fruit (*Passiflora mollissima* (Kunth) L.H. Bailey) using HPLC-DAS-ESI/MS^n^, and determined the antioxidant capacity. HPLC-ESI-MS/MS was employed by Chen et al. [13] to characterize the fingerprint profile of the medicinal herb *Rhamnus davurica* Pall., mostly consisting of flavonoids, which were correlated with the antiproliferative activity of the herbal extracts on different human cancer cell lines.

A particular analytical problem is that raised by polymeric flavan-3-ols (i.e., condensed tannins or proanthocyanidins) that are not well separated by HPLC and cannot be detected as individual compounds. A selective UHPLC-QqQ-MS method using specific MS transitions in MRM mode for compound detection was described by Pinasseau et al. [14] for the characterization and quantification of grape skin flavan-3-ols following depolymerization in the presence of phloroglucinol. In the same paper, the authors also explored the relationship between tannin composition as determined by the proposed method and grape response to drought. Indeed, it is well known that flavonoids play relevant roles in plant physiology and plant ecology, being involved in the natural mechanisms of plant resistance. An interesting immuno-proteomic assay based on the recognition of an antibody against a peptide sequence of rat bilitranslocase was developed by Filippi et al. [15] to identify plant membrane proteins involved in the binding and transport of flavonoids. This approach—which could be extended to other plant proteins with different functions—might be helpful to investigate the role of flavonoids on plant growth and defence against biotic and abiotic stress.

Flavonoids also play a determining role on the stability, sensory, and health properties of foods. Several articles included in this special issue deal with some of those aspects. The effects of different drying methods on polyphenol oxidase (PPO) activity and their influence on the flavonoid and phenolic contents and antioxidant capacity of rhizomes of *Zingiber officinale* var. *rubrum* was studied by Ghasemzadeh et al. [16]. The addition of flavonoid-rich seeds from grape pomace during winemaking was explored by Jara-Palacios et al. [17] as a strategy to improve the antioxidant potential of red wines in a warm climate. Health promoting activities of black crowberry (*Empetrum nigrum* L.)—a relevant source of flavonols and anthocyanins—have been reviewed by Jurikova et al. [18], while physicochemical characteristics, occurrence, and health implications of cyanidin-3-*O*-glucoside (a widespread anthocyanin in food) was the object of the review by Olivas-Aguirre et al. [19].

A variety of biological activities, such as antioxidant, anti-inflammatory, estrogenic, antimicrobial, or antitumor abilities have been reported for flavonoids, and different experimental approaches can be used for their evaluation. A screening method based on immobilized metal affinity chromatography (IMAC) combined with ultrafiltration-UPLC-MS was employed by Zhang et al. [20] to identify ligands for xanthine oxidase in *Gnaphalium hypoleucum* D.C.—a Chinese folk medicine used for the treatment of gout. The angiotensin-converting enzyme (ACE) inhibition activity of flavonoids from *Orthosiphon stamineus* leaves was explored by Shafaei et al. [21]. The authors proposed a method to measure ACE activity based on the determination of hippuric acid formed by the action of the enzyme on an ACE-specific substrate; using molecular docking, they also showed that ACE inhibition by flavonoids was related to their ability to bind Zn(II), further stabilized by interactions with amino acids at the active site of the enzyme. Xanthohumol and related chalcone analogues obtained from hops (*Humulus lupulus*) or prepared by hemisynthesis were studied by Stompor and Zarowska [22] for their antimicrobial activity in an effort to draw conclusions on the structural features associated with their effects on different microorganisms, including Gram-positive and Gram-negative bacteria, fungi, and yeasts.

The molecular mechanisms involved in the apoptotic effects of the two flavonoids nobiletin and myricitrin were studied in different cell lines. Moon and Cho [23] showed that nobiletin was able to induce endoplasmic reticulum (ER) stress-mediated apoptosis and autophagy in SNU-16 cells via the downregulation of Akt/mTOR signalling, whereas Zhang et al. [24] demonstrated that myricitrin activates Nrf2-mediated antioxidant signalling and attenuates H9c2 cell apoptosis induced by high glucose levels via activation of Akt signalling. Studies on animal models were also carried out to evaluate the effects and subjacent mechanisms of the activity of flavonoids on different pathological conditions. Chamorro et al. [25] showed that quercetin prevented the changes in blood pressure and lung injury in a rat model of haemorrhagic shock and reperfusion through inhibition of acid sphingomyelinase activity (aSMase), suggesting that this flavonol might be useful in patients at risk of ischaemia following a haemorrhagic shock, and pointing to sphingolipids as a potential therapeutic target. Using a diet-induced obesity rat model, Huang et al. [26] concluded that soy isoflavones could regulate lipid homeostasis by suppressing the activity of mTORC1 via the Akt signalling pathway, with decreased lipogenesis and adipogenesis and enhanced lipolysis, thus being of potential interest for obesity control. Harasstani et al. [27] observed that a combined treatment with kaempferol and chrysin increased the survival rate of septic mice, and that this favourable effect correlated with an enhancement of their anti-inflammatory and antioxidant activities compared to the individual treatments. Ablat et al. [28] found that a flavonoid-rich extract from safflower exhibited neuroprotective effects in rotenone-induced Parkinson rats, as well as the potential to inhibit apoptosis triggered by neurotoxic species. The treatment reversed the decreased protein expression of tyrosine hydroxylase, dopamine transporter, and DJ-1, and increased the levels of dopamine, whereas it decreased acetylcholine levels, as revealed using magnetic resonance imaging (MRI) techniques.

Most evidence on the biological activity of flavonoids has been obtained in model assays (either in vitro systems or with animals), whilst data on their actual effects in the human body are more scarce. Flavonoids are generally poorly bioavailable and largely biotransformed in the organism. Usually only a small proportion of the consumed flavonoids can be absorbed in the small intestine and are found circulating in the organism in the form of conjugated metabolites; the remaining fraction goes to the large intestine, where the compounds interact with the colonic microflora to produce a variety of low molecular weight metabolites that could be further absorbed and found in blood mostly as conjugated forms. Thus, it might be supposed that the systemic effects of flavonoids would greatly rely on the amounts, distribution, and activity of the produced metabolites. Those aspects are the object of two comprehensive reviews focusing on wine flavonoids. The review by Fernandes et al. [29] pays particular attention to bioavailability, protective effects on different diseases, and molecular mechanisms of action, whereas Cueva et al. [30] lay emphasis on the interactions between polyphenols and gut microbiota and their physiological implications.

The broad range of aspects covered in this *Special Issue* highlights the current interest in flavonoid research in areas such as phytochemistry, organic synthesis, plant and food sciences, and human health. However, rapid advances are expected in the coming years, favoured by the improvements in analytical techniques which will enable the detection, identification, and quantification of new and minor flavonoids in plants and foods and of their metabolites in human fluids and tissues. Among other aspects, this should allow the description of biomarkers for flavonoid consumption and effects, so that some relationships with lower incidence rates of chronic diseases could be established. Since the physicochemical and biological properties of flavonoids are molecule-specific, more structure–activity studies should be done; pure compounds are needed for this, and therefore efficient methods for their synthesis must be developed. Pure compounds should also be submitted to in vitro and in vivo assays to draw conclusions on the molecular mechanisms of action of flavonoids. Furthermore, human intervention trials would be required to delve into the putative beneficial effects of flavonoids on human health. Metabolic engineering approaches could be addressed to increase and/or optimize the flavonoid profiles in plants, so as to improve not only their agronomic characteristics but also the functional and technological properties of edible crops. We hope that the present *Special Issue* will encourage researchers to continue exploring these and other challenges in the fascinating word of flavonoids.
